# Pushing the limits – two new species of *Pteromalus* (Hymenoptera, Chalcidoidea, Pteromalidae) from Central Europe with remarkable morphology

**DOI:** 10.3897/zookeys.514.9910

**Published:** 2015-07-22

**Authors:** Hannes Baur

**Affiliations:** 1 Department of Invertebrates, Natural History Museum Bern, Bernastrasse 15, 3005 Bern, Switzerland; 2 Institute of Ecology and Evolution, University of Bern, Baltzerstrasse 6, 3012 Bern, Switzerland

**Keywords:** Systematics, taxonomy, thorax, morphometry, distance measurements, *Pteromalus apum*, *Pteromalus bifoveolatus*, *Pteromalus cassotis*, *Pteromalus puparum*, *Pteromalus squamifer*, *Pteromalus vanessae*, Pteromalinae, Pireninae, Papilionidae, Pieridae

## Abstract

Two new species, *Pteromalus briani* **sp. n.** and *Pteromalus janstai* **sp. n.**, with unusual characters are described from the Central Plateau and the Alps in Switzerland, respectively. *Pteromalus briani* **sp. n.** is remarkable in that it has the metatibia quite abruptly expanded before the middle. This type of modification of the hind tibia is unique within the Pteromalidae and probably also the entire Chalcidoidea. It is also very rare in other parasitic wasps, where it is suspected to be associated with pheromone glands. The species is a gregarious endoparasitoid of pupae of *Vanessa atalanta* (Linnaeus) and *Aglais urticae* (Linnaeus), two common butterflies (Lepidoptera: Nymphalidae) in Europe. It is furthermore a koinobiont parasitoid ovipositing in an early larval stage of the host. The other species, *Pteromalus janstai* **sp. n.**, shows a flattened mesosoma. A dorsoventrally depressed body is a unique feature within the genus *Pteromalus*, but known from a number species in unrelated genera and subfamilies. The two records demonstrate that it is possible to discover entirely new species with extraordinary characters even in one of the taxonomically most thoroughly explored parts of the world.

## Introduction

In Western Europe the vast majority of newly discovered insect species usually belong to complexes of cryptic species. Morphologically, such new species are therefore quite similar to known species and in many cases even rather difficult to separate from those (e.g., Huber et al. 2013, Alkhatib et al. 2014, Baur et al. 2014, Schmidt et al. 2015). Today the discovery of species with an entirely distinct morphology happens quite rarely and they are then usually found in remote places such as the recently described, spectacular *Cyranobracon depardieui* Quicke and Butcher (Hymenoptera: Braconidae) from tropical Papua New Guinea (Butcher and Quicke 2015) or *Norbanus draco* Mitroiu (Hymenoptera: Pteromalidae) from Central and Southern Africa (Mitroiu 2015). Here I describe two new species of Pteromalidae (Chalcidoidea) with outstanding morphological characters from Central Europe. Although both species are clearly referable to the genus *Pteromalus*, some of their characters stretch the limits of the genus, and in one case the character state may not even be known in Chalcidoidea.

The genus *Pteromalus* contains 485 species world wide, with the majority (371 species) having been described from Europe (Noyes 2015). It is thus the most species-rich genus of Pteromalidae. All species are parasitoids of larvae and pupae of various holometabolous insects, for instance Lepidoptera, Coleoptera, gall forming Hymenoptera (Cynipidae, Tenthredinidae) and Diptera (Tephritidae). No recent study is available that delineates *Pteromalus* based on phylogenetic principals. However, the genus can easily be recognized by a combination of characters (Graham 1969, Bouček and Rasplus 1991, Bouček and Heydon 1997): clypeus striate, its anterior margin truncate or weakly to strongly emarginate, always without a median tooth; flagellum with 2 anelli and 6 funicular segments; clava in females symmetrical; prepectus with relatively small upper triangular area; paraspiracular sulci rather deep and usually with some transverse costulae. *Pteromalus puparum* (Linnaeus, 1758) and *Pteromalus cerealellae* (Ashmead, 1902) are among the best-known species of the genus, while for the majority of species little is known except for an occasional distributional or host record (Noyes 2015). However, some *Pteromalus* species attacking fruit flies (Diptera: Tephritidae) have received attention as potential biological control agents (Kapaun et al. 2010) and in community ecology (e.g., Hoffmeister 1992).

## Material and methods

Specimens are deposited in the following collections (acronyms mostly according to Noyes 2015): The Natural History Museum, London, UK (BMNH); Canadian National Collection, Ottawa, Canada (CNC); Swiss Federal Institute of Technology, Entomology Collection, Zurich, Switzerland (ETHZ); Jacqueline Grosjean, Niederwangen, Switzerland (JGC, private collection); Biological Museum (Entomology), Lund University, Lund, Sweden (LUZM); Muséum d’histoire naturelle, Geneva, Switzerland (MHNG); Natural History Museum, Vienna, Austria (NHMV); Natural History Museum Bern, Bern, Switzerland (NMBE); Staatliches Museum für Naturkunde, Stuttgart, Germany (SMNS); University of Riverside, Riverside, California, USA (UCR); United States National Museum, Washington DC, USA (USNM); Veli Vikberg, Turenki, Finland (VVC, private collection); Zoologisches Forschungsmuseum Alexander Koenig, Bonn, Germany (ZFMK). All specimens were killed with ethyl acetate, mounted on card rectangles following the method described by Noyes (1982), and finally air dried. Some specimens were later dissected for taking photographs.

Geographical coordinates on data labels of type specimens are indicated as WGS 84 latitude and longitude.

Nomenclature and classification of Chalcidoidea follow Noyes (2015). Terminology of body parts follows Gibson (1997), for terms concerning sculpture of the integument and for some particular expressions used in the description I refer to Graham (1969). The separation of the plica of the propodeum into an anterior and a posterior plica is according to Baur (2000). The Appendix gives an overview of the basic descriptive statistics for each body measurement (in µm) and species as well as the sample sizes. The selected measurements correspond to those used in the taxonomy of Pteromalidae for calculating standard ratios (e.g., Graham 1969; see Table 1), except for body length. Body length of Pteromalidae is usually measured in dorsal view from anterior margin of head to tip of ovipositor sheaths (Graham 1969, Bouček and Rasplus 1991). It is thus often quite variable due to the varying position and angle of head and gaster relative to mesosoma. I therefore have preferred to indicate body length as the sum of lengths (in mm) of head, mesosoma and gaster, each of which could be measured rather more precisely. I also give mesoscutum breadth (in µm), which is considered by Ohl and Thiele (2007) as the most universal measure of size in some Apoidea (Hymenoptera). Note that such a measure is the best way to compare the size of females and males in Chalcidoidea, since body length is usually strongly affected by sex related differences of the gaster (see Bouček 1988, Gibson et al. 1997).

**Table 1.** Abbreviation, name, definition, and magnification of the 41 measurements used in the description (see Material and methods for further information).

Abbreviation	Character name	Definition	Magnification in pixel/mm*
ant.l	Pedicel plus flagellum length	Combined length of pedicel plus flagellum, outer aspect (Graham 1969)	1742
clv.b	Clava breadth	Greatest breadth of clava, outer aspect	3910
clv.l	Clava length	Greatest length of clava, outer aspect	3910
eye.b	Eye breadth	Greatest breadth of eye, lateral view	3910
eye.d	Eye distance	Shortest distance between eyes, dorsal view	1742
eye.h	Eye height	Greatest length of eye height, lateral view	2549
eye.l	Eye length	Length of eye, dorsal view (Graham 1969)	888
fl3.b	First funicular segment breadth	Greatest breadth of first funicular segment (= third flagellar segment), outer aspect	3910
fl3.l	First funicular segment length	Greatest length of first funicular segment (= third flagellar segment), outer aspect	3910
fl8.b	Sixth funicular segment breadth	Greatest breadth of sixth funicular segment (= eighth flagellar segment), outer aspect	3910
fl8.l	Sixth funicular segment length	Greatest length of sixth funicular segment (= eighth flagellar segment), outer aspect	3910
fm3.b	Metafemur breadth	Greatest breadth of metafemur, outer aspect	3910
fm3.l	Metafemur length	Length of metafemur, from distal end of trochanter to tip of metafemur, measured along midline, outer aspect	1742
fwi.b	Fore wing breadth	Greatest breadth of fore wing, measured at about right angle to marginal and postmarginal veins	1742/1089
fwi.l	Fore wing length	Greatest length of fore wing, measured from end of humeral plate to tip of wing	1089
gst.b	Gaster breadth	Greatest breadth of gaster, distance between the outermost lateral edges of the gaster, dorsal view	1742/2549
gst.l	Gaster length	Length of gaster along median line from posterior edge of nucha to tip of ovipositor sheath, dorsal view	1089
hea.b	Head breadth	Greatest breadth of head, dorsal view	1742
hea.h	Head height	Distance between anterior margin of clypeus and anterior edge of anterior ocellus, frontal view	1742
hea.l	Head length	Length of head, dorsal view (Graham 1969)	888
lof.h	Lower face height	Distance between anterior margin of clypeus and lower margin of torulus	2549
mav.l	Marginal vein length	Length of marginal vein, distance between the point at which the submarginal vein touches the leading edge of the wing and the point at which stigmal vein and postmarginal vein unite (Graham 1969)	2549
msc.b	Mesoscutum breadth	Greatest breadth of mesoscutum just in front of level of tegula, dorsal view	1742
msc.l	Mesoscutum length	Length of mesoscutum along median line from posterior edge of pronotum to posterior edge of mesoscutum, dorsal view	2549
msp.l	Malar space	Distance between the point where malar sulcus enters mouth margin and malar sulcus enters lower edge of eye, lateral view (Graham 1969)	3910
mss.l	Mesosoma length	Length of mesosoma along median line from anterior edge of pronotum collar to posterior edge of nucha, dorsal view	1089
ool.l	OOL	Shortest distance between posterior ocellus and eye margin, dorsal view (Graham 1969)	3910
pdl.b	Pedicel breadth	Greatest breadth of pedicel, outer aspect	3910
pdl.l	Pedicel length	Length of pedicel, outer aspect	3910
plc.d	Plica distance	Greatest distance between upper edge of anterior plica	2549
pmv.l	Postmarginal vein length	Length of postmarginal vein (Graham 1969), distance between the point at which the stigmal vein and postmarginal vein unite, apically to where the vein appears to end	2549
pol.l	POL	Shortest distance between posterior ocelli, dorsal view (Graham 1969)	3910
ppd.l	Propodeum length	Length of propodeum measured along median line from anterior edge to posterior edge of nucha, dorsal view	2549
scp.b	Scape breadth	Greatest breadth of scape, outer aspect	3910
scp.l	Scape length	Length of scape exclusive of radicle, outer aspect (Graham 1969)	2549
sct.l	Scutellum length	Length of scutellum along median line from posterior edge of mesoscutum to posterior edge of scutellum, dorsal view	2549
stv.l	Stigmal vein length	Length of stigmal vein, distance between the point at which stigmal vein and postmarginal vein unite apically, and the distal end of the stigma (Graham 1969)	2549
ta3.l	Metatarsus length	Length of metatarsus, including pretarsus	2549
tb3.b	Metatibia breadth	Apical breadth of metatibia, outer aspect	3910
tb3.l	Metatibia length	Length of metatibia, measured along midline, outer aspect	1742
tmp.l	Temple length	Length of temple, dorsal view (Graham 1969)	888

* Where more than one number is present, the first refers to the magnification used for *Pteromalus briani* sp. n., the second to the one used for *Pteromalus janstai* sp. n.

Most characters were measured on photographs taken by Lisa Wilmsmeier with a Leica DFC425 camera mounted on a Leica M16 stereomicroscope. Photographs were taken at different magnifications depending on the size of the character, in order to reduce measurement error for the smaller ones. For all measurements, it was ensured that the points of reference were in perfect focus and that the diaphragm of the lens was fully open. The distances were finally measured using ImageJ, version 1.46v (Schneider et al. 2012). Body parts on the images were zoomed-in on screen up to four times before measuring. To avoid variation due to fluctuating asymmetry (e.g., Palmer and Strobeck 1986, Bechshøft et al. 2008), measurements of paired characters were taken on the left hand side.

I measured three characters, eye length, head length, and temple length, on a single stack photograph taken with a Keyence VHX 2000 digital photomicroscope and a VH-Z20R/W zoom lens at a magnification of 200× (i.e., 1000 µm corresponded to 888 pixels, see Table 1), also using ImageJ, version 1.48v. Stack photos were used because the reference points do not lie in the same focal plane. Accuracy of measurement is thus critically dependent on an exact positioning of the head in dorsal view. Naturally, measurement error should be higher for such characters.

I also used the Keyence microscope for making stack-images of qualitative character states. A 4-digit individual code including the notion “Baur” (e.g., “Baur 2410”) was provided for specimens that have been measured or photographed, or used as reference specimens for comparison with newly described species.

## Data resources

I compiled all morphological data in a FileMaker Pro 12®, version 12.0v5, database, of which natural language descriptions as well as ranges of body ratios were generated using the FileMaker script language. Because this is commercial software, a qualitative and a quantitative data matrix (raw values in µm) were exported as comma separated values (CSV) files made available at the BMNH data portal at DOI: http://dx.doi.org/10.5519/0056966. The repository furthermore contains all photographs used for measurements, photographs of reference specimens (sometimes provided by other institutions, see acknowledgments) and of labels of the holotypes of the newly described species.

## Results

### Pteromalus briani sp. n.

Taxon classificationAnimaliaHymenopteraPteromalidae

http://zoobank.org/58D10F28-31F6-4E6C-AC8C-90FFBDA10ADC

Figs 1 , 2A, B

#### Type material.

Holotype ♀ Switzerland, Canton Bern, Köniz, Niederwangen, 570 m, 46.92361°N, 7.37266°E, leg. Jacqueline Grosjean, 28-ii-2004, ex pupa 16-iii-2004, host *Vanessa atalanta* (Linnaeus, 1758) (Lepidoptera: Nymphalidae), deposited in NMBE (Baur 2129). The host pupa was collected in sheltered cavity of a pedestrian underpass beneath the highway and railway line in Niederwangen. Paratypes 46 ♀ 2 ♂, emerging from the same host pupa as the holotype, deposited in: 2 ♀ BMNH, 2 ♀ CNC, 2 ♀ ETHZ, 5 ♀ JGC, 2 ♀ LUZN, 2 ♀ MHNG, 2 ♀ NHMV, 19 ♀ (Baur 2408, 2414, 2416, 2418–2421, 2423–2426) 2 ♂ (Baur 2139, 2415) NMBE, 2 ♀ SMNS, 2 ♀ UCR, 2 ♀ USNM, 2 ♀ VVC, 2 ♀ ZFMK. Paratypes 6 ♀ Switzerland, Canton Bern, Reichenbach, Kien, 560 m, 46.6132°N, 7.6854°E, v-2008, leg. Rahel Schnidrig, reared from pupa of *Aglais urticae* (Linnaeus, 1758) (Lepidoptera: Nymphalidae), deposited in: 1 ♀ CNC, 5 ♀ NMBE. According to Schnidrig (pers. comm.), the host was collected as a larva (size about 2.5 mm) and afterwards reared under protected conditions. A total of 40–50 specimens emerged from the pupa but only the paratypes were preserved.

#### Description, female.

*Color*: Head and mesosoma: green to blue-green with metallic luster; setae on head and mesosoma: whitish, inconspicuous; tegula: testaceous; setae on callus of propodeum: whitish.

Scape: testaceous; pedicel: testaceous, slightly infuscate dorsally; flagellum: brown.

Fore wing: hyaline; fore wing venation: testaceous; setae on fore wing: fuscous; hind wing: hyaline.

Coxae: green; trochanters: testaceous; femora: testaceous; tibiae: testaceous; tarsi: testaceous with fifth segment slightly infuscate; pretarsi: slightly infuscate.

Petiole: green with purplish tinge; gaster: green; gastral terga: one to five with strong purplish tinge.

**Figure 1.**
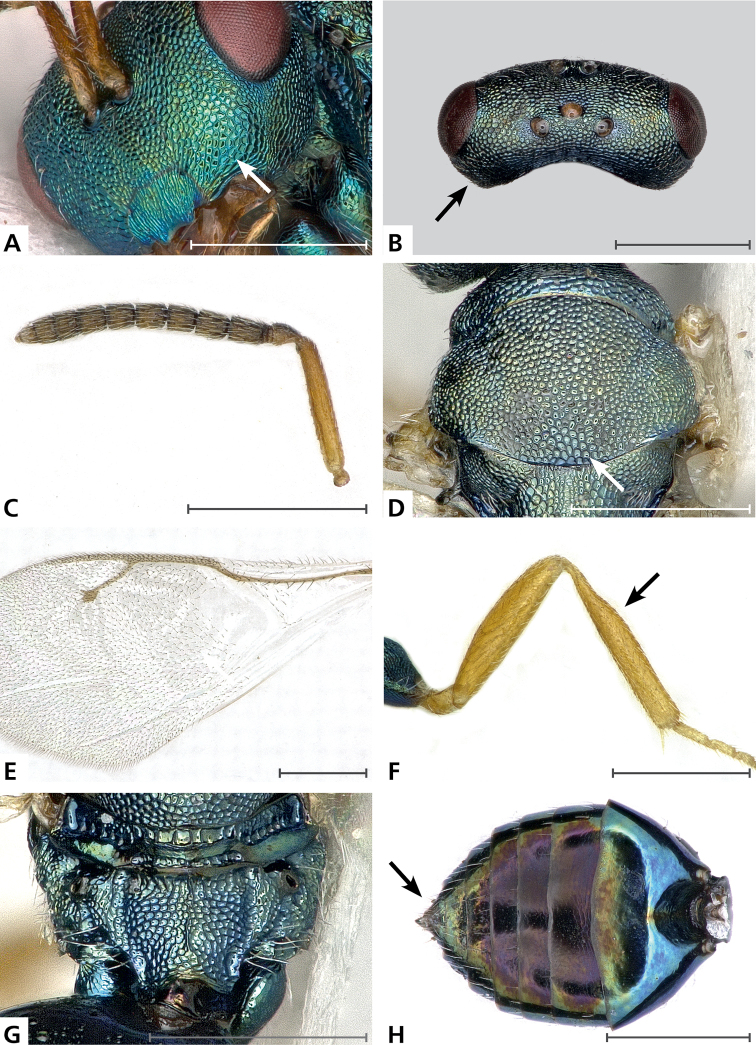
**A**, **D**, **G** *Pteromalus briani* sp. n. holotype ♀, **B**, **C**, **E**, **F**, **H** paratype ♀. **A** gena, anterolateral view **B** head, dorsal view **C** left antenna, outer aspect **D** mesoscutum, dorsal view **E** fore wing venation **F** left metatibia, outer aspect **G** propodeum, dorsal view **H** gaster, dorsal view. Arrows mark important character states; scale bars 0.5 mm.

*Sculpture*: Head in frontal view: finely reticulate with relatively high dividing septa; clypeus: finely striate (Fig. 1A); area between clypeus and malar sulcus: meshes of reticulation conspicuously enlarged (Fig. 1A).

Mesoscutum: finely reticulate, meshes rather high, areoles small and only moderately enlarged in posterior part of sclerite (Fig. 1D); scutellum: reticulate, meshes about as strong and coarse as on posterior part of mesoscutum, but with a narrow band of smaller areoles in anterior half of median longitudinal line; frenum: reticulate, meshes of similar size to those on scutellum; axilla: reticulate, about as strong as on central part of scutellum; prepectus upper triangular area: reticulate; upper mesepimeron: anteriorly smooth, posterior corner distinctly alutaceous; upper mesepisternum: reticulate, about as strong as on mesoscutum; metapleuron: reticulate, about as strong as on mesepisternum.

Pro- and mesocoxa: finely alutaceous, metacoxa: finely reticulate.

Median area of propodeum: evenly reticulate, as strong as on mesoscutum (Fig. 1G); inner corner of anterior plica: with a depression, weakly reticulate; nucha: reticulate, as strong as on median area of propodeum; callus of propodeum: reticulate; paraspiracular sulcus: reticulate with few transverse costulae.

Petiole in dorsal view: smooth; gastral terga: smooth and shining, sixth tergum and syntergum alutaceous (Fig. 1H).

*Shape and structure*: Head in frontal view: subtrapezoid; gena in frontal view: rounded; temple in dorsal view: obtuse (Fig. 1B); forming an angle with occiput of: 120 degrees; occipital carina: absent; torulus position with respect to lower ocular line: distinctly above; lower face in lateral view: flat, receding with respect to upper face: weakly, forming an angle of: 35 degrees; scrobe: narrow, rather shallow; malar sulcus: superficial, but traceable; clypeus, anterior margin: widely and shallowly emarginate, without a slight depression above emarginate edge; gena near mouth: terete; tentorial pit: distinctly visible (Fig. 1A); mouth extension: not conspicuously enlarged; mandibular formula: 4-4.

Antenna (Fig. 1C). Antennal formula: 11263; scape reaching: distinctly above level of vertex; flagellum: filiform; first anellus: strongly transverse; second anellus: strongly transverse; first funicular segment: cylindrical; setae on flagellum: moderately thickly clothed with setae standing out at an angle of 30 degrees, length of setae less than half the breadth of flagellar segments; number of rows of longitudinal sensilla on first funicular segment: 2, on sixth: 1–2.

Mesosoma in lateral view: moderately strongly bent; propodeum in lateral view sloping with respect to dorsal plane of mesoscutum and scutellum at an angle of: 45 degrees; pronotum breadth with respect to mesoscutum breadth: distinctly narrower; pronotum collar: horizontal, well defined, its length with respect to mesoscutum length: one sixth, its anterior margin: rounded edge; pronotum posterior margin: thin, shiny strip; notaulus: extremely superficial, hardly traceable, reaching: about half along mesoscutum (Fig. 1D); scutellum in lateral view: moderately convex; scutellum in posterior view: moderately convex; scutellum posterior margin projection: level of anterior margin of dorsellum; scutellum posterior margin in posterior view: narrowly emarginate in the middle; frenal line: finely indicated, especially on sides; prepectus upper triangular area: not separated by oblique carina; upper mesepimeron: strongly narrowing below, not reaching base of mesopleuron; propodeum (Fig. 1G): anterior plica: bent inwards in anterior two fifths and strong; posterior plica: present, joining or almost joining anterior plica; orientation of posterior plicae: almost parallel; median carina of propodeum: weakly indicated, irregular; nucha: elevated but not clearly differentiated from median area of propodeum; spiracle: oval, size: small, separated from anterior margin of propodeum by: shortest diameter; callus pilosity: numerous long setae; paraspiracular sulcus: narrow and deep.

Fore wing (Fig. 1E). Fore wing apex with respect to apex of gaster when folded back: distinctly exceeding; basal cell number of setae: 7; basal setal line: complete, with: 6 setae; cubital setal line: incomplete, with: 4 setae; costal cell pilosity on dorsal side: bare; costal cell pilosity on lower side: with numerous setae in distal half and a complete setal line extending to base; speculum on upper side: bare, widely open below; fore wing disc: rather thickly pilose; marginal setae: present, short; stigma: subcircular, small; uncus: short.

Femora: moderately slender; metatibia: quite abruptly expanded before the middle (Fig. 1F); metacoxa pilosity, dorsally: bare.

Petiole in dorsal view: conical, in ventral view: open; gaster in dorsal view: ovate, obtusely pointed (Fig. 1G); gastral terga: weakly sunken; posterior margin of first gastral tergum: slightly curved backwards medially; first gastral tergum reaching: two fifths of gaster; tip of hypopygium reaching: slightly beyond middle of gaster; ovipositor sheath: slightly protruding.

*Length and body ratios*: Body length: 2.3–2.9 mm; mesoscutum breadth: 591–806 µm.

Head breadth to height: 1.2–1.41; head breadth to length: 2.02–2.08; head breadth to mesoscutum breadth: 1.26–1.34; lower face height to head height: 0.5–0.58; POL to OOL: 0.76–0.87; eye height to breadth: 1.3–1.36; eye distance to height: 1.74–1.88; temple length to eye length: 0.35–0.44; malar space to eye height: 0.68–0.76.

Pedicel plus flagellum length to head breadth: 0.72–0.87; scape length to eye height: 0.99–1.04; scape length to breadth: 5.24–5.82; pedicel length to breadth: 1.22–1.54; pedicel length to first funicular segment length: 0.84–1.13; first funicular segment length to breadth: 0.91–1.33; sixth funicular segment length to breadth: 0.85–1.04; first funicular segment breadth to clava breadth: 0.85–1.06; clava length to breadth: 2.01–2.57.

Mesosoma length to mesoscutum breadth: 1.5–1.6; mesoscutum breadth to length: 1.57–1.76; mesoscutum length to scutellum length: 1.03–1.1; propodeum length to scutellum length: 0.57–0.62; plica distance to propodeum length: 1.21–1.31.

Fore wing length to breadth: 2–2.18; marginal vein length to stigmal vein length: 1.51–1.68; postmarginal vein length to stigmal vein length: 0.78–1.01.

Metafemur length to breadth: 3.27–4.47; metatibia length to breadth: 5.61–7.82; metatarsus length to metatibia length: 0.65–0.89.

Gaster length to breadth: 1.17–1.62; gaster length to mesosoma length: 0.82–1.11.

#### Description, male.

*Color*: Head and mesosoma: bright green to blue-green with metallic luster; setae on head and mesosoma: whitish, inconspicuous; tegula: testaceous; setae on callus of propodeum: whitish.

Scape: testaceous; pedicel: testaceous, slightly infuscate dorsally; flagellum: testaceous, slightly infuscate dorsally.

Fore wing: hyaline; fore wing venation: testaceous; setae on fore wing: fuscous; hind wing: hyaline.

Coxae: green; trochanters: testaceous; femora: testaceous; tibiae: testaceous; tarsi: testaceous with fifth segment slightly infuscate; pretarsi: slightly infuscate.

Petiole: green with purplish tinge; gaster: green; gastral terga: one to three with an indistinct yellowish spot.

**Figure 2.**
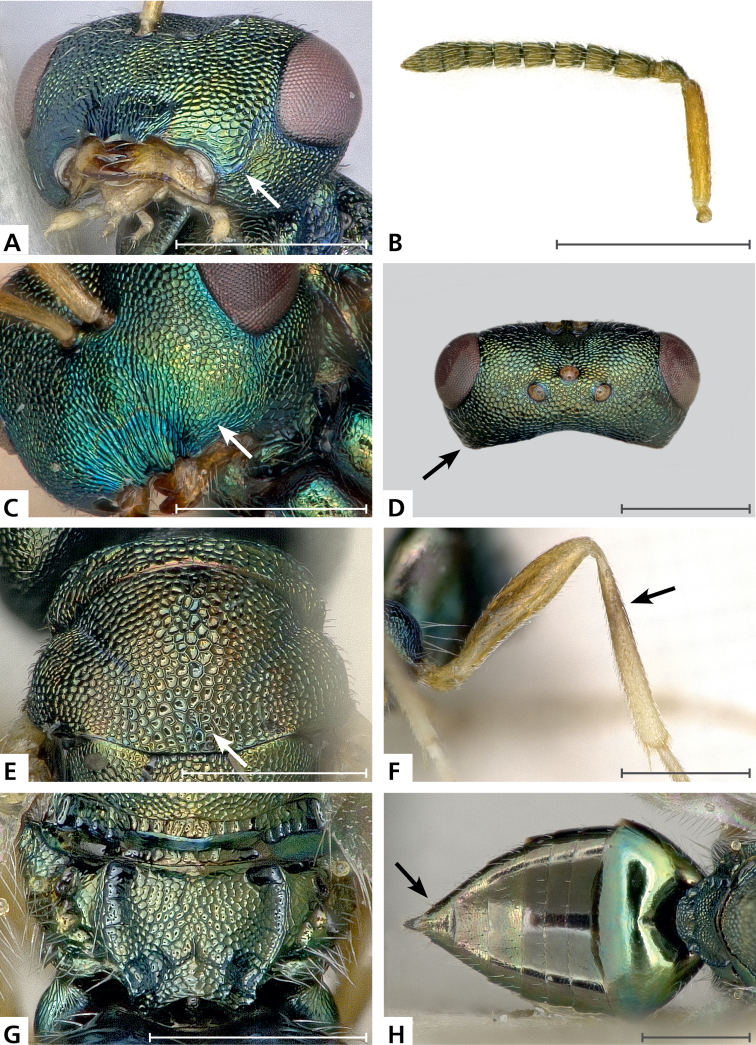
**A**, **B** *Pteromalus briani* sp. n. paratype ♂, **C**–**H** *Pteromalus squamifer* Thomson ♀, from Sweden. **A** head, ventral view **B** left antenna, outer aspect **C** gena, anterolateral view **D** head, dorsal view **E** mesoscutum, dorsal view **F** left metatibia, outer aspect **G** propodeum, dorsal view **H** gaster, dorsal view. Arrows mark important character states; scale bars 0.5 mm.

*Sculpture*: Head in frontal view: finely reticulate with relatively high septae; clypeus: finely striate; area between clypeus and malar sulcus: meshes conspicuously enlarged (Fig. 2A).

Mesoscutum: finely reticulate, meshes rather high, areoles small and only moderately enlarged in posterior part of sclerite; scutellum: reticulate, meshes about as strong and coarse as on posterior part of mesoscutum, but with a narrow band of smaller areoles in anterior half of median longitudinal line; frenum: reticulate, meshes of similar size to those on scutellum; axilla: reticulate, about as strong as on central part of scutellum; prepectus upper triangular area: reticulate; upper mesepimeron: anteriorly smooth, posterior corner distinctly alutaceous; upper mesepisternum: reticulate, about as strong as on mesoscutum; metapleuron: reticulate, about as strong as on mesepisternum.

Pro- and mesocoxa: finely alutaceous, metacoxa: finely reticulate.

Median area of propodeum: evenly reticulate, as strong as on mesoscutum; inner corner of anterior plica: with a depression, weakly reticulate; nucha: reticulate, as strong as on median area of propodeum; callus of propodeum: reticulate; paraspiracular sulcus: reticulate with few transverse costulae.

Petiole in dorsal view: smooth; gastral terga: smooth and shining, sixth tergum and syntergum alutaceous.

*Shape and structure*: Head in frontal view: subtrapezoid; gena in frontal view: rounded; temple in dorsal view: obtuse; forming an angle with occiput of: 120 degrees; occipital carina: absent; torulus position with respect to lower ocular line: distinctly above; lower face in lateral view: flat, receding with respect to upper face: weakly, forming an angle of: 35 degrees; scrobe: narrow, rather shallow; malar sulcus: superficial, but traceable; clypeus, anterior margin: widely and shallowly emarginate, without a median depression above emarginate edge; gena near mouth: terete; tentorial pit: distinctly visible; mouth extension: not conspicuously enlarged (Fig. 2A); mandibular formula: 4-4.

Antenna (Fig. 2B). Antennal formula: 11263; scape reaching: distinctly above level of vertex; flagellum: filiform; first anellus: strongly transverse; second anellus: strongly transverse; setae on flagellum: thickly clothed with setae standing out at an angle of 40 degrees, length of setae less than half the breadth of flagellar segments; number of rows of longitudinal sensilla on first funicular segment: 1, on sixth: 1.

Mesosoma in lateral view: moderately strongly bent; propodeum in lateral view sloping with respect to dorsal plane of mesoscutum and scutellum at an angle of: 50 degrees; pronotum breadth with respect to mesoscutum breadth: distinctly narrower; pronotum collar: horizontal, well defined, its length with respect to mesoscutum length: one sixth, its anterior margin: rounded edge; pronotum posterior margin: thin, shiny strip; notaulus: extremely superficial, hardly traceable, reaching: about half along mesoscutum; scutellum in lateral view: moderately convex; scutellum in posterior view: moderately convex; scutellum posterior margin projection: level of anterior margin of dorsellum; scutellum posterior margin in posterior view: narrowly emarginate in the middle; frenal line: finely indicated, especially on sides; prepectus upper triangular area: separated by a fine oblique carina; upper mesepimeron: strongly narrowing below, not reaching base of mesopleuron; anterior plica: bent inwards in anterior two fifths and strong; posterior plica: present, joining anterior plica; orientation of posterior plicae: almost parallel; median carina of propodeum: weakly indicated, irregular; nucha: elevated but not clearly differentiated from median area of propodeum; spiracle: oval, size: small, separated from anterior margin of propodeum by: shortest diameter; callus pilosity: numerous long setae; paraspiracular sulcus: narrow and deep.

Fore wing apex with respect to apex of gaster when folded back: distinctly exceeding; basal cell number of setae: 6; basal setal line: complete, with: 8 setae; cubital setal line: incomplete, with: 4 setae; costal cell pilosity on dorsal side: bare; costal cell pilosity on lower side: numerous setae in distal half and a complete setal line extending to base; speculum on upper side: bare, widely open below; fore wing disc: rather thickly pilose; marginal setae: present, short; stigma: subcircular, small; uncus: short.

Femora: moderately slender; metatibia: quite abruptly expanded before the middle; metacoxa pilosity, dorsally: bare.

Petiole in dorsal view: conical, in ventral view: open; gaster in dorsal view: ovate; gastral terga: weakly sunken.

*Length and body ratios*: Body length: 2.7 mm; mesoscutum breadth: 682–684 µm.

Head breadth to height: 1.46–1.47; head breadth to length: 2.02–2.03; head breadth to mesoscutum breadth: 1.3; lower face height to head height: 0.59–0.6; POL to OOL: 0.89–0.96; eye height to breadth: 1.29–1.3; eye distance to height: 1.78; temple length to eye length: 0.39–0.43; malar space to eye height: 0.58–0.61.

Pedicel plus flagellum length to head breadth: 0.84; scape length to eye height: 0.97–0.98; scape length to breadth: 4.89–5.15; pedicel length to breadth: 1.37; pedicel length to first funicular segment length: 0.85–0.96; first funicular segment length to breadth: 1.27–1.57; sixth funicular segment length to breadth: 1.02–1.05; first funicular segment breadth to clava breadth: 0.91–0.98; clava length to breadth: 2.44–3.13.

Mesosoma length to mesoscutum breadth: 1.63–1.64; mesoscutum breadth to length: 1.48–1.5; mesoscutum length to scutellum length: 1.08–1.12; propodeum length to scutellum length: 0.55–0.59; plica distance to propodeum length: 1.21–1.39.

Fore wing length to breadth: 2–2.02; marginal vein length to stigmal vein length: 1.39–1.56; postmarginal vein length to stigmal vein length: 0.84–0.93.

Metafemur length to breadth: 4.23–4.62; metatibia length to breadth: 7.16–7.35; metatarsus length to metatibia length: 0.72–0.74.

Gaster length to breadth: 1.68–1.71; gaster length to mesosoma length: 1–1.01.

#### Comment.

Close examination of the expanded metatibia under a stereomicroscope did not reveal any distinctive characteristics compared to the “normal”, i.e. unexpanded, metatibia of the other *Pteromalus* species. It should be noted that for some of the specimens reared from *Aglais urticae* the expansion is slightly less abrupt than shown in Fig. 1F.

#### Diagnosis.

The female of *Pteromalus briani* sp. n. keys out in Graham (1969) via couplets 1, 2, 7–9, 11, 12, 14, 49, 52–57, 88–90 (alternatively couplets 49, 70, 72, 74, 78, 79, 84, 88–90) to *Pteromalus smaragdus* Graham. The male keys out via couplets 1–3, 5, 7, 10, 11, 14–19, 40, 44, 45, 54–56, 65 to *Pteromalus semotus* and *Pteromalus varians* [sub *Pteromalus grandis*]. The species belongs to a group of species with 4 teeth in both mandibles and a large reticulate nucha (i.e., to *Pteromalus* sensu stricto of Graham 1969). In this group it is most similar to *Pteromalus puparum* and *Pteromalus squamifer*, especially in the structure of the propodeum (compare Figs 1G and 2G). It is distinguished from those and all other species by the following combination of characters: female legs except coxae bright testaceous (Fig. 1F); reticulation between clypeus and malar sulcus with enlarged meshes (Fig. 1A, 2A); POL distinctly less than OOL (Fig. 1B); tentorial pit distinctly visible (Fig. 1A); antenna inserted high on face, lower edge of torulus above the middle between anterior margin of clypeus and anterior edge of anterior ocellus; mesoscutum with areoles small and only moderately enlarged in posterior part of sclerite (Fig. 1D); scutellum in lateral view moderately convex; metatibia quite abruptly expanded before the middle (Fig. 1F); female gaster obtusely pointed (Fig. 1H), usually less than 1.6 times as long as broad.

Below the most important differences are given for those species with which *Pteromalus briani* sp. n. might be most easily confounded. Because of the difficulty to identify some of them, a rather large number of taxa either related to *Pteromalus puparum* or with similar hosts (Lepidoptera: Papilionidae, Nymphalidae or Pieridae) has been considered.

*Pteromalus apum* (Retzius, 1783): female femora infuscate; reticulation between clypeus and malar sulcus without enlarged meshes; POL greater than OOL; tentorial pit indistinct; antenna inserted less high on face, lower edge of torulus below the middle between anterior margin of clypeus and anterior edge of anterior ocellus; mesoscutum with areoles small and only moderately enlarged in posterior part of sclerite; scutellum in lateral view flattened; metatibia gradually widening towards apex; female gaster acuminate, often more than 1.6 times as long as broad. Source of information: 2 ♀ 2 ♂ from Switzerland in NMBE (Baur 2517–2520), also compared with the key by Askew and Shaw (1997).

*Pteromalus bifoveolatus* (Förster, 1861): female femora infuscate; reticulation between clypeus and malar sulcus without enlarged meshes; POL slightly greater than OOL; tentorial pit indistinct; antenna high on face, lower edge of torulus at about the middle between anterior margin of clypeus and anterior edge of anterior ocellus; mesoscutum with areoles small and only moderately enlarged in posterior part of sclerite; scutellum in lateral view moderately convex; metatibia gradually widening towards apex; female gaster acuminate, often more than 1.6 times as long as broad. In addition, the male of *Pteromalus bifoveolatus* is special in that the mouth is very wide, so that the malar space is much less than 0.1 times as long as eye height (0.58–0.61 in *Pteromalus briani* sp. n.). Source of information: syntype ♂ in NHMV, 2 ♀ 2 ♂ (Baur 2521–2524) from Switzerland in NMBE.

*Pteromalus cassotis* Walker, 1847 (syn. *Pteromalus archippi* Howard, 1889: 1891): female legs except coxae testaceous; reticulation between clypeus and malar sulcus without enlarged meshes; POL about as great as OOL; tentorial pit indistinct; antenna high on face, lower edge of torulus at about the middle between anterior margin of clypeus and anterior edge of anterior ocellus; mesoscutum with areoles small and only moderately enlarged in posterior part of sclerite; scutellum in lateral view moderately convex; metatibia gradually widening towards apex; female gaster acuminate, about 1.25 times as long as broad. Source of information: photographs of lectotype ♀ in BMNH, provided by N. Dale-Skey Papilloud; lectotype ♀ of *Pteromalus archippi* in USNM.

*Pteromalus fuscipes* (Provancher, 1881): The lectotype is deposited in the Laval University, Quebec, Canada (Noyes 2015; Huber, pers. comm.), but was not available for examination. The original description (see Provancher 1881: 295) suggests a species with dark legs (“Pattes brunes” = legs brown), which naturally excludes an identity with *Pteromalus briani* sp. n. Burks (1963: 1262) suggested that *Pteromalus fuscipes* might be the same as *Pteromalus puparum vanessae* (see also below).

*Pteromalus luzonensis* Gahan, 1925: female femora infuscate; reticulation between clypeus and malar sulcus without enlarged meshes; POL about as great as OOL; tentorial pit indistinct; antenna high on face, lower edge of torulus at about the middle between anterior margin of clypeus and anterior edge of anterior ocellus; mesoscutum with areoles small and only moderately enlarged in posterior part of sclerite; scutellum in lateral view moderately convex; metatibia gradually widening towards apex; female gaster obtusely pointed, 1.4–1.6 times as long as broad. Source of information: photographs of a syntype ♀ from Luzon, Mount Makiling, provided by the USNM Chalcidoidea type catalog. 5 ♀ 5 ♂ from Assam and Nepal, in BMNH, compared with the original description by Gahan (1925: 99–100).

*Pteromalus melitaeae* Dzhanokmen, 1998: female femora infuscate; reticulation between clypeus and malar sulcus without enlarged meshes; POL greater than OOL; tentorial pit indistinct; antenna less high on face, lower edge of torulus slightly below the middle between anterior margin of clypeus and anterior edge of anterior ocellus; mesoscutum with areoles small and only moderately enlarged in posterior part of sclerite; scutellum in lateral view moderately convex; metatibia gradually widening towards apex; female gaster acuminate, about 2.3 times as long as broad. Source of information: 2 ♀ from Switzerland in NMBE (Baur 2525, 2526), compared with a paratype 1 ♀ in BMNH and the English version of the original description by Dzhanokmen (1998).

*Pteromalus platensis* Brèthes *in* Massini, 1913 (syn. *Pteromalus caridei* Brèthes, 1913: 93, synonymized by De Santis 1967: 197): The name-bearing types are not available for examination (Noyes 2015). The descriptions of *Pteromalus platensis* and *Pteromalus caridei* (see Massini 1913: 517, Brèthes 1913: 93, and Massini and Brèthes 1918, 2. plate), suggest a species with dark femora close to *Pteromalus puparum*, which thus excludes it from being the same as *Pteromalus briani* sp. n.

*Pteromalus platyphilus* Walker, 1874: female femora infuscate; reticulation between clypeus and malar sulcus without enlarged meshes; POL greater than OOL; tentorial pit indistinct; antenna less high on face, lower edge of torulus distinctly below the middle between anterior margin of clypeus and anterior edge of anterior ocellus; mesoscutum with areoles small and only moderately enlarged in posterior part of sclerite; scutellum in lateral view moderately convex; metatibia gradually widening towards apex; female gaster obtusely pointed, about 1.3 times as long as broad. Source of information: 1 ♀ from Morocco in NMBE (Baur 2527), det. Z. Bouček 1996.

*Pteromalus puparum* (Linnaeus, 1758): female femora infuscate; reticulation between clypeus and malar sulcus without enlarged meshes; POL slightly greater than OOL; tentorial pit indistinct; antenna high on face, lower edge of torulus at about the middle between anterior margin of clypeus and anterior edge of anterior ocellus; mesoscutum with areoles small and only moderately enlarged in posterior part of sclerite; scutellum in lateral view moderately convex; metatibia gradually widening towards apex; female gaster obtusely pointed, rarely more than 1.6 times as long as broad. Source of information: 3 ♀ 2 ♂ from Switzerland in NMBE (Baur 2528–2531, 2549).

*Pteromalus puparum vanessae* Howard, 1889: Harris (1841: 220–221) originally proposed the specific name “Pteromalus vanessae” but without accompanying description. Hence it has to be considered as a nomen nudum (Noyes 2015). Howard (1889: 1891–1892) who gave a brief description based on material reared from *Nymphalis antiopa* (Linnaeus, 1758) (sub *Euvanessa antiopa*) and *Polygonia interrogationis* (Fabricius, 1798) (both Lepidoptera: Nymphalidae), eventually made the name available. The whereabouts of the syntypes is unknown (Noyes 2015) and they thus could not be checked. However, Howard (1889) evidently considered *Pteromalus puparum vanessae* to be only a larger and darker variety of *Pteromalus puparum*, of which he gave a redescription (p. 1890). The latter is said to have dark legs, which differentiates the species from *Pteromalus archippi* (= *Pteromalus cassotis*, see above) with pale legs described by Howard in the same paper (p. 1891). Therefore, *Pteromalus puparum vanessae* also must have dark legs, which clearly separates it from *Pteromalus briani* sp. n.

*Pteromalus semotus* (Walker, 1834): female femora infuscate; reticulation between clypeus and malar sulcus without enlarged meshes; POL distinctly greater than OOL; tentorial pits indistinct; antenna less high on face, lower edge of torulus slightly below the middle between anterior margin of clypeus and anterior edge of anterior ocellus; mesoscutum with areoles small and only moderately enlarged in posterior part of sclerite; scutellum in lateral view moderately convex; metatibia gradually widening towards apex; female gaster acuminate, distinctly more than twice as long as broad. Source of information: 1 ♀ from Switzerland in NMBE (Baur 2532), compared with the lectotype ♀ in BMNH.

*Pteromalus smaragdus* Graham, 1969: female legs except coxae bright testaceous [this is in contrast to the original description, where it is stated on p. 494 that the legs have the same color as *Pteromalus procerus* (Graham, 1969) which is said to have the femora infuscate (p. 493)]; reticulation between clypeus and malar sulcus without enlarged meshes; POL slightly greater than OOL; tentorial pit indistinct; antenna high on face, lower edge of torulus at about the middle between anterior margin of clypeus and anterior edge of anterior ocellus; mesoscutum with areoles small and only moderately enlarged in posterior part of sclerite; scutellum in lateral view moderately convex; metatibia gradually widening towards apex; female gaster acuminate, about 1.3 times as long as broad. Source of information: photographs of holotype ♀ in BMNH, provided by N. Dale-Skey Papilloud.

*Pteromalus squamifer* (Thomson, 1878): female legs except coxae testaceous (Fig. 2F); reticulation between clypeus and malar sulcus without enlarged meshes (Fig. 2C); POL slightly less than OOL (Fig. 2D); tentorial pit indistinct (Fig. 2C); antenna high on face, lower edge of torulus at about the middle between anterior margin of clypeus and anterior edge of anterior ocellus; mesoscutum with areoles large and rather strongly enlarged in posterior part of sclerite (Fig. 2E); scutellum in lateral view moderately convex; metatibia gradually widening towards apex (Fig. 2F); female gaster acuminate (Fig. 2H), 1.55–1.6 times as long as broad. As in *Pteromalus bifoveolatus*, the male has the mouth very large (see Graham 1969: 399, figure 338) and malar space much less than 0.1 times as long as eye height (0.58–0.61 in male *Pteromalus briani* sp. n., Fig. 2A). Source of information: photographs of lectotype ♀ in LUZM, provided by C. Hansson; 1 ♀ from Italy in NMBE (Baur 2533) and 4 ♀ from Sweden in BMNH (Baur 2545–2548). It should be noted that in the key of Graham (1969: 513–514) couplet 91 to *Pteromalus squamifer* might be misleading, in that he stated “temples about two thirds as long as eyes”. In fact, my measurements on a photograph as well as on the other specimens showed that the temple is at most 0.6 times as long as the eye (Fig. 1C). This value is also strongly depending on how the head is positioned. In another photograph after re-positioning of the same specimen, the ratio was only 0.5!

*Pteromalus varians* (Spinola, 1808): female femora varying from infuscate to testaceous; reticulation between clypeus and malar sulcus without enlarged meshes; POL distinctly greater than OOL; tentorial pits indistinct; antenna high on face, lower edge of torulus at about the middle between anterior margin of clypeus and anterior edge of anterior ocellus; mesoscutum with areoles small and only moderately enlarged in posterior part of sclerite; scutellum in lateral view moderately convex; metatibia gradually widening towards apex; female gaster acuminate, distinctly more than twice as long as broad. Source of information: 4 ♀ 1 ♂ from France, Moldavia, and Switzerland in NMBE (Baur 2534–2539), compared with lectotypes of synonyms of *Pteromalus varians*, that is, ♀ *Pteromalus grandis* Walker, 1835 and ♀ *Pteromalus latipennis* Walker, 1835 in BMNH.

*Pteromalus vopiscus* Walker, 1839: female femora infuscate; reticulation between clypeus and malar sulcus without enlarged meshes; POL slightly greater than OOL; tentorial pit indistinct; antenna high on face, lower edge of torulus at about the middle between anterior margin of clypeus and anterior edge of anterior ocellus; mesoscutum with areoles small and only moderately enlarged in posterior part of sclerite; scutellum in lateral view moderately convex; metatibia gradually widening towards apex; female gaster acuminate, often more than 1.6 times as long as broad. Source of information: 2 ♀ from Switzerland, in NMBE (Baur 2540, 2541). Identification originally based on Graham’s (1995) redescription of the species, however, the specimens were later also compared with specimens from Southern France in BMNH identified by Graham himself.

#### Etymology.

Following the suggestion of the collector of the new species, Jacqueline Grosjean, *Pteromalus briani* sp. n. is named after Brian Jones, since the *Vanessa atalanta* pupa was collected on his birthday. The name “briani” is a noun in the genitive case and need not agree in gender with the generic name.

#### Biology.

*Pteromalus briani* sp. n. is a gregarious, primary endoparasitoid of pupae of Nymphalidae (Lepidoptera). Currently, *Vanessa atalanta* and *Aglais urticae* are known as hosts but the species is likely to attack pupae of other nymphalids or possibly of related families. About 58–60 specimens emerged from the overwintering pupa of *Vanessa atalanta* (only 51 ♀, 2 ♂ preserved). According to Rahel Schnidrig (pers. com.) about 40–50 specimens emerged from the pupa of *Aglais urticae* but only 6 ♀ were preserved. The investigation of Schnidrig suggests a koinobiont life history strategy, because the host was collected in an early larval stage (body length 2.5 mm), which was afterwards protected from further parasitization during captive rearing.

### Pteromalus janstai sp. n.

Taxon classificationAnimaliaHymenopteraPteromalidae

http://zoobank.org/856D795F-691C-41EE-9E54-89FA67700253

Fig. 3

#### Type material.

Holotype ♀ Switzerland, Canton Wallis, Kippel, Zend, 2100 m, 46.4069°N, 7.7494°E, 15.07.2005, leg. P. Jansta & H. Baur, 15-vii-2005, on Larch (*Larix decidua* Mill.), NMBE (Baur 2410). Paratype 1 ♀, same data as holotype, BMNH (Baur 2411). Paratype 1 ♂ Switzerland, Canton Grisons, Samedan, Blais Granda, 2100 m, 46.4412°N, 9.86456°E, 10-viii-1998, leg. H. Baur, NMBE (Baur 2412).

#### Description, female.

*Color*: Head and mesosoma: green to blue-green with metallic luster; setae on head and mesosoma: fuscous, inconspicuous; tegula: green; setae on callus of propodeum: whitish.

Scape: fuscous with basal third testaceous; pedicel: fuscous; flagellum: fuscous.

Fore wing: hyaline; fore wing venation: brownish; setae on fore wing: fuscous; hind wing: hyaline.

Coxae: green; trochanters: slightly greenish, testaceous at tips; pro- and mesofemur: green, testaceous in apical quarter, metafemur: green, testaceous in apical sixth; protibia: testaceous, meso- and metatibia: testaceous, medially slightly infuscate; tarsi: testaceous, apical segments slightly infuscate; pretarsi: slightly infuscate.

Petiole: dark purplish; gaster: green to blue-green with metallic luster; gastral terga: one to five with strong purplish tinge.

*Sculpture*: Head in frontal view: finely reticulate with moderately high septae; clypeus: striate; area between clypeus and malar sulcus: finely reticulate.

Mesoscutum: finely reticulate, meshes moderately high, areoles small and not enlarged in posterior part of sclerite; scutellum: reticulate, meshes about as strong and coarse as on posterior part of mesoscutum; frenum: reticulate, meshes larger than those on scutellum; axilla: reticulate, about as strong as on central part of scutellum; prepectus upper triangular area: weakly reticulate; upper mesepimeron: anteriorly smooth, posterior corner distinctly alutaceous; upper mesepisternum: reticulate, about as strong than on mesoscutum; metapleuron: weakly reticulate, less strong as on mesepisternum.

Coxae: weakly reticulate.

Median area of propodeum: uniformly reticulate, as strong as on mesoscutum; inner corner of anterior plica: with a smooth depression and transverse carinae; nucha: reticulate, as strong as on median area of propodeum; callus of propodeum: weakly reticulate; paraspiracular sulcus: smooth with few transverse costulae.

Petiole in dorsal view: smooth; gastral terga: smooth and shining, third to fifth tergum anteriorly, sixth tergum and syntergum wholly alutaceous.

**Figure 3.**
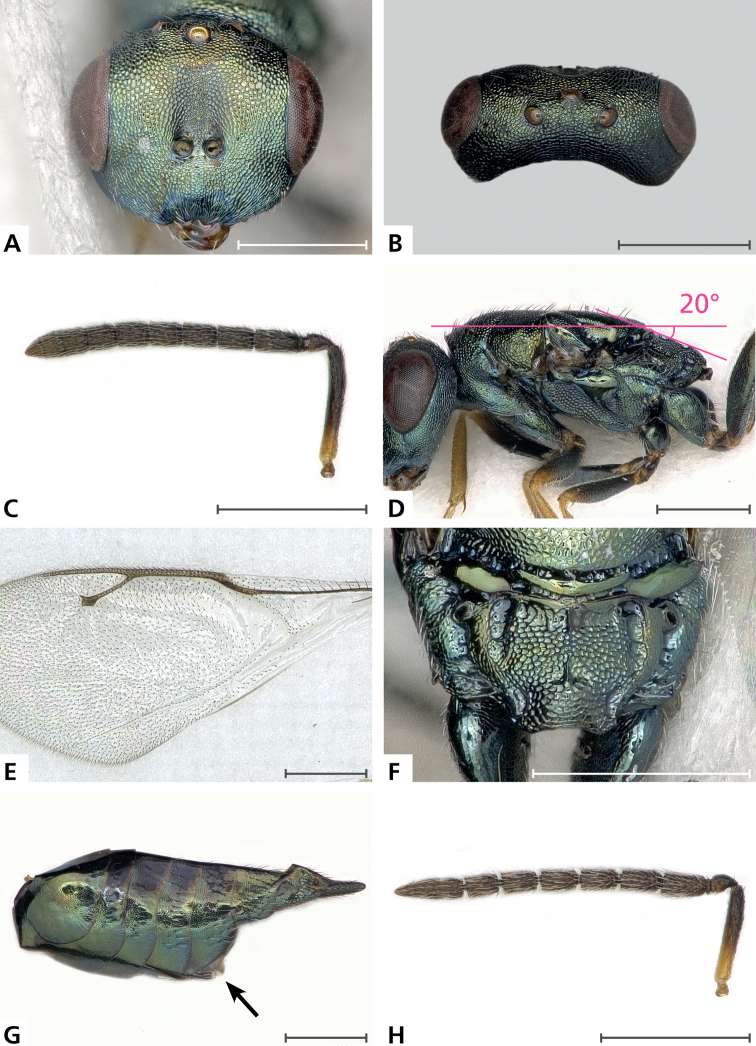
**A**–**G** *Pteromalus janstai* sp. n. paratype ♀, **H** paratype ♂. **A** head, frontal view **B** head, dorsal view **C** left antenna, outer aspect ♀ **D** mesosoma, lateral view **E** fore wing venation **F** propodeum, dorsal view **G** gaster, lateral view **H** left antenna, outer aspect ♂. Arrows mark important character states; scale bars 0.5 mm.

*Shape and structure*: Head in frontal view: subtrapezoid (Fig. 3A); gena in frontal view: buccate; temple in dorsal view: obtuse (Fig. 3B); forming an angle with occiput of: 110 degrees; occipital carina: absent; torulus position with respect to lower ocular line: above; lower face in lateral view: weakly curved, receding with respect to upper face: weakly, forming an angle of: 35 degrees; scrobe: narrow, moderately deep; malar sulcus: superficial, but traceable; clypeus, anterior margin: widely and shallowly emarginate, medially slightly inclined above anterior edge (Fig. 3A); gena near mouth: terete; tentorial pit: indistinct (Fig. 3A); mouth extension: not conspicuously enlarged (Fig. 3A); mandibular formula: 3-4.

Antenna (Fig. 3C). Antennal formula: 11263; scape reaching: middle of anterior ocellus; flagellum: almost filiform; first anellus: strongly transverse; second anellus: strongly transverse; first funicular segment: very slightly constricted at base; setae on flagellum: moderately thickly clothed with setae standing out at an angle of 10–20 degrees, length of setae less than half the breadth of flagellar segments; number of rows of longitudinal sensilla on first funicular segment: 2, on sixth: 1–2.

Mesosoma in lateral view: rather flattened; propodeum in lateral view sloping with respect to dorsal plane of mesoscutum and scutellum at an angle of: 20 degrees (Fig. 3D); pronotum breadth with respect to mesoscutum breadth: distinctly narrower; pronotum collar: horizontal, well defined, its length with respect to mesoscutum length: one sixth, its anterior margin: finely carinate; pronotum posterior margin: thin, shiny strip; notaulus: superficial, reaching: two thirds along mesoscutum; scutellum in lateral view: almost flat; scutellum in posterior view: almost flat medially; scutellum posterior margin projection: level of anterior margin of dorsellum; scutellum posterior margin in posterior view: straight; frenal line: finely indicated, especially on sides; prepectus upper triangular area: separated by a strong carina; upper mesepimeron: strongly narrowing below, not reaching base of mesopleuron; propodeum (Fig. 3F): anterior plica: present, almost straight in anterior part; posterior plica: present, joining or almost joining anterior plica; orientation of posterior plicae: almost parallel; median carina of propodeum: mostly effaced; nucha: elevated but not clearly differentiated from median area of propodeum; spiracle: oval, size: small, separated from anterior margin of propodeum by: shortest diameter; callus pilosity: relatively sparsely setose; paraspiracular sulcus: narrow and deep.

Fore wing (Fig. 3E). Fore wing apex with respect to apex of gaster when folded back: just reaching; basal cell number of setae: 9–12 setae in distal part; basal setal line: complete, with: 11–12 setae; cubital setal line: incomplete, with: 4–8 setae; costal cell pilosity on dorsal side: bare; costal cell pilosity on lower side: with numerous setae in distal half and one setal line extending to base; speculum on upper side: bare, widely open below; fore wing disc: moderately thickly pilose; marginal setae: present, short; stigma: subrectangular, small; uncus: short.

Femora: slender; metatibia: gradually widening towards apex; metacoxa pilosity, dorsally: bare.

Petiole in dorsal view: conical, in ventral view: open; gaster in dorsal view: very elongate and acuminate; gastral terga: strongly convex; posterior margin of first gastral tergum: entire; first gastral tergum reaching: one fourth of gaster; tip of hypopygium reaching: almost three fifths of gaster (Fig. 3G); ovipositor sheath: distinctly protruding.

*Length and body ratios*: Body length: 3.9–4 mm; mesoscutum breadth: 815–829 µm.

Head breadth to height: 1.35–1.39; head breadth to length: 2.08–2.1; head breadth to mesoscutum breadth: 1.18; lower face height to head height: 0.42–0.44; POL to OOL: 1.19–1.2; eye height to breadth: 1.54–1.65; eye distance to height: 1.4–1.46; temple length to eye length: 0.36–0.45; malar space to eye height: 0.45–0.48.

Pedicel plus flagellum length to head breadth: 1.05–1.07; scape length to eye height: 0.81–0.86; scape length to breadth: 6.05–6.39; pedicel length to breadth: 1.51; pedicel length to first funicular segment length: 0.64–0.68; first funicular segment length to breadth: 1.77–1.83; sixth funicular segment length to breadth: 1.06–1.1; first funicular segment breadth to clava breadth: 0.8–0.84; clava length to breadth: 2.09–2.24.

Mesosoma length to mesoscutum breadth: 1.7–1.71; mesoscutum breadth to length: 1.44–1.52; mesoscutum length to scutellum length: 1.13–1.22; propodeum length to scutellum length: 0.59–0.61; plica distance to propodeum length: 1.2–1.36.

Fore wing length to breadth: 2.24–2.3; marginal vein length to stigmal vein length: 1.7–1.78; postmarginal vein length to stigmal vein length: 0.93–0.99.

Metafemur length to breadth: 3.88–4.47; metatibia length to breadth: 7.19–7.29; metatarsus length to metatibia length: 0.8.

Gaster length to breadth: 5.04–5.35; gaster length to mesosoma length: 1.51–1.52.

#### Description, male.

*Color*: Head and mesosoma: bright green to blue-green with metallic luster; setae on head: whitish, inconspicuous, on mesosoma: whitish, inconspicuous; tegula: green; setae on callus of propodeum: whitish.

Scape: fuscous with basal two fifths testaceous; pedicel: fuscous; flagellum: brown.

Fore wing: hyaline; fore wing venation: brownish testaceous; setae on fore wing: fuscous; hind wing: hyaline.

Coxae: green; pro- and mesotrochanter: slightly infuscate, metatrochanter: fuscous; pro- and mesofemur: infuscate, testaceous in apical third, metafemur: green, testaceous on tips; tibiae: testaceous; protarsus: slightly infuscate, meso- and metatarsus: testaceous, apical segments slightly infuscate; pretarsi: slightly infuscate.

Petiole: dark purplish; gaster: green; gastral terga: basal terga with large dark yellow spot.

*Sculpture*: Head in frontal view: finely reticulate with moderately high septae; clypeus: striate; area between clypeus and malar sulcus: finely reticulate.

Mesoscutum: finely reticulate, meshes moderately high, areoles small and not enlarged in posterior part of sclerite; scutellum: weakly reticulate, meshes less strong and coarse than on posterior part of mesoscutum; frenum: weakly reticulate, meshes larger than those on scutellum; axilla: reticulate, about as strong as on lateral part of scutellum; prepectus upper triangular area: weakly reticulate; upper mesepimeron: anteriorly smooth, posterior corner distinctly alutaceous; upper mesepisternum: reticulate, about as strong as on mesoscutum; metapleuron: weakly reticulate, less strong than on mesepisternum.

Pro- and mesocoxa: finely alutaceous, metacoxa: finely reticulate.

Median area of propodeum: uniformly reticulate, as strong as on mesoscutum but with smaller meshes; inner corner of anterior plica: with a smooth depression and transverse carinae; nucha: reticulate, as strong as on median area of propodeum; callus of propodeum: weakly reticulate; paraspiracular sulcus: smooth with few transverse costulae.

Petiole in dorsal view: smooth; gastral terga: smooth and shining, second to sixth tergum and syntergum alutaceous.

*Shape and structure*: Head in frontal view: subtrapezoid; gena in frontal view: buccate; temple in dorsal view: obtuse; occipital carina: absent; torulus position with respect to lower ocular line: distinctly above; lower face in lateral view: rather flat, receding with respect to upper face: weakly, forming an angle of: 35 degrees; scrobe: narrow, moderately deep; malar sulcus: superficial, but traceable; clypeus, anterior margin: widely and shallowly emarginate, medially slightly inclined above anterior edge; gena near mouth: terete; tentorial pit: indistinct; mouth extension: not conspicuously enlarged; mandibular formula: ?3-4 (the mandibles are in the single male concealed, but the mandibular formula is most likely the same as in females).

Antenna (Fig. 3H). Antennal formula: 11263; scape reaching: posterior edge of anterior ocellus; flagellum: filiform; first anellus: strongly transverse; second anellus: strongly transverse; first funicular segment: slightly conical; setae on flagellum: thickly clothed with setae standing out at an angle of 50–60 degrees, length of setae slightly shorter than half the breadth of flagellar segments; number of rows of longitudinal sensilla on first funicular segment: 1, on sixth: 1.

Mesosoma in lateral view: rather flattened; propodeum in lateral view sloping with respect to dorsal plane of mesoscutum and scutellum at an angle of: about 25 degrees; pronotum breadth with respect to mesoscutum breadth: distinctly narrower; pronotum collar: horizontal, well defined, its length with respect to mesoscutum length: one sixth, its anterior margin: slightly elevated edge, medially carinate; pronotum posterior margin: thin, shiny strip; notaulus: superficial, reaching: two thirds along mesoscutum; scutellum in lateral view: almost flat; scutellum in posterior view: almost flat medially; scutellum posterior margin projection: level of anterior margin of dorsellum; scutellum posterior margin in posterior view: narrowly emarginate in the middle; frenal line: finely indicated, especially on sides; prepectus upper triangular area: ? (the lower part of the prepectus is concealed in the single male, but the character state is likely to be the same as for the females); upper mesepimeron: strongly narrowing below, not reaching base of mesopleuron; anterior plica: present, almost straight in anterior part; posterior plica: present, joining anterior plica; orientation of posterior plicae: almost parallel; median carina of propodeum: anteriorly indicated, effaced posteriorly; nucha: elevated but not clearly differentiated from median area of propodeum; spiracle: oval, size: small, separated from anterior margin of propodeum by: shortest diameter; callus pilosity: relatively sparsely setose; paraspiracular sulcus: narrow and deep.

Fore wing apex with respect to apex of gaster when folded back: not exceeding; basal cell number of setae: with up to 10 setae in distal part; basal setal line: complete, with: 11 setae; cubital setal line: incomplete, with: 4 setae; costal cell pilosity on dorsal side: bare; costal cell pilosity on lower side: with numerous setae in distal half and a complete setal line extending to base; speculum on upper side: bare, widely open below; fore wing disc: moderately thickly pilose; marginal setae: present, short; stigma: subrectangular, small; uncus: short.

Femora: slender; metatibia: gradually widening towards apex; metacoxa pilosity, dorsally: bare.

Petiole in dorsal view: conical, in ventral view: open; gaster in dorsal view: elongate, obtuse; gastral terga: weakly sunken; posterior margin of first gastral tergum: entire; first gastral tergum reaching: slightly less than one third of gaster.

*Length and body ratios*: Body length: 3.1 mm; mesoscutum breadth: 732 µm.

Head breadth to height: 1.44; head breadth to length: 2.06; head breadth to mesoscutum breadth: 1.17; lower face height to head height: 0.51; POL to OOL: 1.33; eye height to breadth: 1.39; eye distance to height: 1.46; temple length to eye length: 0.38; malar space to eye height: 0.44.

Pedicel plus flagellum length to head breadth: 1.3; scape length to eye height: 0.84; scape length to breadth: 5.42; pedicel length to breadth: 1.28; pedicel length to first funicular segment length: 0.54; first funicular segment length to breadth: 2.08; sixth funicular segment length to breadth: 1.41; first funicular segment breadth to clava breadth: 0.97; clava length to breadth: 3.25.

Mesosoma length to mesoscutum breadth: 1.65; mesoscutum breadth to length: 1.44; mesoscutum length to scutellum length: 1.23; propodeum length to scutellum length: 0.64; plica distance to propodeum length: 1.1.

Fore wing length to breadth: 2.11; marginal vein length to stigmal vein length: 1.75; postmarginal vein length to stigmal vein length: 0.91.

Metafemur length to breadth: 4.3; metatibia length to breadth: 7.04; metatarsus length to metatibia length: 0.79.

Gaster length to breadth: 3.38; gaster length to mesosoma length: 1.19.

#### Comment.

The dorsoventrally compressed mesosoma and the shape of the propodeum allowed an easy association of the females with the male even though they were collected in separate localities (about 160 km as the crow flies).

#### Diagnosis.

*Pteromalus janstai* sp. n. is distinguished from all known species of *Pteromalus* species by the following combination of characters: mesosoma strongly flattened; female gaster elongate, laterally strongly compressed, more than 5 times as long as broad.

The female keys out in Graham (1969) via couplets 1, 2, 7–9, 11, 12, 14, 49, 52–56, 58, 59, 60, 62 to couplet 63, where it fits neither of the two species, *Pteromalus dispar* (see below) and “H. sp. indet. C”. The male keys out via couplets 1–3, 5, 7, 10, 11, 14–19, 40, 44, 50, 52, 53, 54, 55, 56, 57, and 59 where both options don’t match well.

Most similar are the following species but they differ – among many other characters mentioned in the description – by a rather more strongly bent mesosoma and a much less elongate female gaster:

*Pteromalus cyniphidis* (Linnaeus, 1758) (syn. *Pteromalus capreae* (Linnaeus, 1761)), *Pteromalus dispar* (Curtis, 1827), *Pteromalus dolichurus* (Thomson, 1878), *Pteromalus fasciatus* (Thomson, 1878), *Pteromalus pontaniae* (Askew, 1985) and *Pteromalus tereus* Walker, 1839. Source of information, beside the keys of Graham (1969) and Askew (1995): *Pteromalus dispar* 2 ♀ 1 ♂ from Denmark and Switzerland in NMBE (Baur 2542–2544); specimens compared with material identified by Graham and Bouček as well as lectotypes of synonyms of *Pteromalus dispar*, that is, ♀ *Pteromalus mesochlorus* Walker, 1835, ♀ *Pteromalus saravus* Walker, 1845, ♀ *Pteromalus basalis* Walker, 1835, and ♂ *Pteromalus cabarnos* Walker, 1839 in BMNH. Lectotype ♀ of *Pteromalus dolichurus* and of *Pteromalus fasciatus* in LUZM, and lectotype ♂ of *Pteromalus tereus* in BMNH. Furthermore, Veli Vikberg kindly compared photographs of *Pteromalus janstai* sp. n. with specimens of *Pteromalus cyniphidis* in VVC. Some of these specimens belong to the same reared series from which the neotype ♂ of *Pteromalus cyniphidis* was selected by Vikberg and Askew (2006). Vikberg (pers. comm.) confirmed that the two species are clearly separated by the mentioned characters.

#### Etymology.

*Pteromalus janstai* sp. n. is named after Petr Jansta, who collected the female specimens. The name “janstai” is a noun in the genitive case and need not agree in gender with the generic name.

#### Biology.

Host unknown. The females of *Pteromalus janstai* sp. n. were swept on some isolated Larch trees (*Larix decidua*) in an Alpine meadow. The male was swept in a similar habitat, but it cannot be determined whether it was swept from trees.

## Discussion

Although the two new species are clearly placed within the genus *Pteromalus*, their morphology and some life history traits are remarkable and merit discussion. The most notable morphological feature concerns the metatibia of *Pteromalus briani* sp. n. Its abrupt expansion in proximal half is unique, not only within the genus but also within the family Pteromalidae and – as far as I can judge – the entire Chalcidoidea. Expansions of tibiae are known from some Pteromalidae, but here they look quite different. For instance, in *Spathopus* (Pireninae) the metatibia is conspicuously but very uniformly swollen only in males. Furthermore, the mesotibia of males of some *Mesopolobus*, *Pegopus*, and *Spaniopus* (Pteromalinae) differs in that the expansion is accompanied by a flattening or at least lateral compression of the tibia (Graham 1969, Bouček 1972). In the case of *Mesopolobus* the mesotibia also shows some special processes and coloration (Graham 1969, Mitroiu 2010). While such an ornamentation may play a role in courtship or during mating (Assem 1974; reviewed by Wehling 1986), a possible behavioral function of the expanded metatibia in *Pteromalus briani* sp. n. remains unknown.

The expansion of the metatibia is a very rare phenomenon in parasitoid wasps. Quicke and Falco (1998) have reported for *Vipio moneilemae* Gahan, 1930 a putative pheromone gland associated modification, which they assumed is unique within the Braconidae. Here, the swelling is present only in males and the dorsal side of the tibia has a longitudinal groove bordered by lateral ridges. In *Pteromalus briani* sp. n., the expansion is the same in both sexes and is not accompanied by a structural modification of the integument. However, only the use of scanning electron microscopy and histological serial sections of fresh material could possibly reveal the structure and function of this particular character. Special attention should be paid to the presence of metatibial glands, such as those found in some aculeate Hymenoptera (Hölldobler et al. 1996).

The rearing of the host larva under protected condition suggests that *Pteromalus briani* sp. n. develops as a gregarious, koinobiont endoparasitoid, since the host was allowed to continue its development after oviposition in an early larval stage and was only killed in the pupal stage. This is in contrast to some related gregarious endoparasitoids of Lepidoptera pupa. For instance, *Pteromalus puparum*, a widespread parasitoid of Papilionidae and Pieridae, immobilizes the pupal stage of its host on which the development also takes place (Takagi 1985, 1986, 1987). This species thus shows an idiobiont life history strategy (Fortuna et al. 2012).

The other species, *Pteromalus janstai* sp. n., is unique within *Pteromalus* because of its flattened mesosoma. This trait is reported from species of a number of other genera of Pteromalidae, for instance *Macroglenes* (Pireninae), *Anogmus*, *Guancheria*, *Monoksa*, *Pachyneuron*, *Platypteromalus*, *Psilonotus*, *Rakosina*, *Syntomopus*, and *Zdenekiana* (Pteromalinae) (Graham 1969, Bouček and Rasplus 1991), but also from various other families, e.g., some species of *Baryscapus* and *Pronotalia* (Eulophidae) (Graham 1987, 1991). In certain families of Chalcidoidea, like the Encyrtidae and Aphelinidae, a flattened body characterizes most species. The function of the flattening remains unclear in a particular case. In Pteromalinae, species of genera, which are more closely related to *Pteromalus* (e.g., *Anogmus*, *Psilonotus*), are often parasitoids of gall midges (Diptera: Cecidomyiidae) (Noyes 2015), and perhaps the trait could be indicative for the unknown host of *Pteromalus janstai* sp. n.

## Supplementary Material

XML Treatment for Pteromalus briani

XML Treatment for Pteromalus janstai

## Appendix

**Appendix:** Overview of 41 measurements (in µm) of *Pteromalus briani* sp. n. and *Pteromalus janstai* sp. n., showing minimum, maximum, mean, and standard deviation (except for *Pteromalus janstai* male with n = 1). For character name and definition, see Table 1.

	*Pteromalus briani*, females, n = 11				*Pteromalus briani*, males, n = 2				*Pteromalus janstai*, females, n = 2				*Pteromalus janstai*, male, n = 1
Character	MIN	MAX	MEAN	SD	MIN	MAX	MEAN	SD	MIN	MAX	MEAN	SD	VALUE
ant.l	577	815	749.2	73.72	743	743	743.4	0.01	1033	1035	1033.9	0.93	1116
clv.b	65	90	77.2	6.88	64	75	69.3	8.23	94	97	95.4	1.64	72
clv.l	168	197	184	8.31	183	199	190.9	10.9	201	211	206.1	6.58	235
eye.b	237	304	280.4	28.08	272	274	273.4	1.47	291	299	295.2	5.42	282
eye.d	559	739	673.6	79.6	631	631	631.2	0.34	668	674	671.2	4.3	572
eye.h	315	408	371.3	38.59	355	355	355	0.01	459	481	470	15.25	393
eye.l	231	301	276	28.51	262	267	264.9	3.57	291	297	293.9	4.8	280
fl3.b	68	84	74	4.86	62	69	65.4	4.82	77	79	78.4	1.5	70
fl3.l	63	110	88.4	15.64	87	97	92.3	6.99	140	141	140.7	0.55	146
fl8.b	65	83	75.5	5.85	64	65	64.7	1.03	81	85	83	3.15	71
fl8.l	63	82	73.3	6.22	65	69	67	2.61	85	94	89.8	6.18	101
fm3.b	115	155	138.6	17	128	137	132.6	5.85	151	175	163.1	17.35	134
fm3.l	496	659	581.7	70.88	578	594	586.2	11.28	675	681	677.7	3.99	577
fwi.b	794	1054	961	110.3	903	905	903.8	1.32	1118	1142	1130.1	17.32	928
fwi.l	1721	2214	2002.6	208.5	1813	1825	1818.8	8.59	2564	2568	2565.9	2.79	1963
gst.b	622	875	765.9	91.68	650	673	661.4	16.26	403	415	409.1	8.2	424
gst.l	932	1111	1045.7	46.68	1113	1133	1123.1	14.28	2090	2157	2123.4	47.45	1435
hea.b	789	1036	946.4	102.7	886	889	887.8	1.96	965	981	972.8	11.33	859
hea.h	575	740	688.1	60.65	604	610	607.1	3.76	694	727	710.4	23.5	598
hea.l	380	507	461.5	53.02	436	440	437.8	2.81	459	472	465.5	9.54	416
lof.h	333	423	388.8	36.03	355	367	361.1	8.45	303	303	303	0.5	304
mav.l	349	475	419.8	48.08	362	381	371.4	12.89	533	557	545	16.8	433
msc.b	591	806	731	87.75	682	684	683.3	1.43	815	829	822	9.83	732
msc.l	363	511	451.8	61.49	456	462	458.9	4.39	536	577	556.7	29.11	509
msp.l	222	299	262.4	27.02	205	217	211.1	8.98	214	219	216.4	3.43	171
mss.l	940	1279	1140.1	136.82	1118	1118	1118.3	0.32	1384	1419	1401.7	24.4	1208
ool.l	170	236	211.3	25.8	187	189	187.9	1.93	185	188	186.3	2.03	153
pdl.b	53	67	62.5	5.33	61	61	60.9	0.52	59	63	61.1	2.45	61
pdl.l	69	97	86.5	10.33	83	84	83.4	0.99	90	95	92.2	3.68	79
plc.d	256	358	314.9	40.58	299	318	308.5	13.17	345	380	362.6	25.06	294
pmv.l	301	414	364.9	38.59	320	339	329.3	13.34	518	530	524.2	8.42	392
pol.l	142	186	167.2	17.67	168	179	173.5	7.83	222	224	222.9	0.97	202
ppd.l	202	286	251.1	30.68	228	248	238.3	14.05	279	288	283.4	6.24	267
scp.b	59	78	69.2	6.93	67	71	69	3.07	61	65	62.8	3.12	61
scp.l	320	412	375.9	37.81	344	348	346	2.7	387	393	390.2	4.27	329
sct.l	346	463	421.5	51.33	413	422	417.5	6.71	474	475	474.3	0.55	414
stv.l	215	293	263.8	32.12	244	260	251.9	11.59	314	314	313.9	0.41	248
ta3.l	400	529	485.5	48.7	468	478	473.1	7.13	672	675	673.4	2.51	572
tb3.b	78	110	98.5	10.45	88	91	89.6	2.31	115	117	116.1	0.94	102
tb3.l	586	778	696.5	80.08	646	653	649.8	4.77	840	842	840.9	0.83	721
tmp.l	84	130	108.8	18.49	103	113	107.8	6.77	107	130	118.6	15.93	107
